# Design of a Ka-Band Five-Bit MEMS Delay with a Coplanar Waveguide Loaded U-Shaped Slit

**DOI:** 10.3390/mi14081508

**Published:** 2023-07-27

**Authors:** Yongxin Zhan, Yu Chen, Honglei Guo, Qiannan Wu, Mengwei Li

**Affiliations:** 1 School of Instrument and Electronics, North University of China, Taiyuan 030051, Chinalmwprew@163.com (M.L.); 2 Academy for Advanced Interdisciplinary Research, North University of China, Taiyuan 030051, China; 3 Center for Microsystem Intergration, North University of China, Taiyuan 030051, China; 4 School of Instrument and Intelligent Future Technology, North University of China, Taiyuan 030051, China; 5 School of Semiconductors and Physics, North University of China, Taiyuan 030051, China; 6 Key Laboratory of Dynamic Measurement Technology, North University of China, Taiyuan 030051, China

**Keywords:** SP6T RF MEMS switch, triangular upper electrode, CPW loading gaps, high delay accuracy, low insertion loss, high-frequency band

## Abstract

This paper designs a five-bit microelectromechanical system (MEMS) time delay consisting of a single-pole six-throw (SP6T) RF switch and a coplanar waveguide (CPW) microstrip line. The focus is on the switch upper electrode design, power divider design, transmission line corner compensation structure design, CPW loading U-shaped slit structure design, and system simulation. The switch adopts a triangular upper electrode structure to reduce the cantilever beam equivalent elastic coefficient and the closed contact area to achieve low drive voltage and high isolation. The SP6T RF MEMS switch uses a disc-type power divider to achieve consistent RF performance across the output ports. When designed by loading U-shaped slit on transmission lines and step-compensated tangents at corners, the system loss is reduced, and the delay amount is improved. In addition, the overall size of the device is 2.1 mm × 2.4 mm × 0.5 mm, simulation results show that the device has a delay amount of 0–60 ps in the frequency range of 26.5–40 GHz, the delay accuracy at the center frequency is better than 0.63 ps, the delay error in the whole frequency band is less than 22.2%, the maximum insertion loss is 3.69 dB, and the input–output return rejection is better than 21.54 dB.

## 1. Introduction

Currently, the wireless communications market is highly competitive, and tiny, low-cost, reconfigurable RF modules are a major research hotspot. Delayers are the essential components of phased array antennas, mainly used in radar systems [1]. Digital delay techniques require the sampling of signals, normally requiring analog-to-digital converters (ADC) [2], therefore leading to relatively high power consumption, quantization noise, and sampling confusion, in addition to the inclusion of ADCs causing problems such as clock injection, nonlinearity, and bandwidth limitations, which can be avoided by using analog delay circuits [3]. Conventional analog delayers use PIN diodes as signal conduction and disconnection elements, but such transistor switching introduces significant losses, up to 8–9 dB at 35 GHz [4]. Instead, it becomes a performance-limiting component of the system. Compared with the delayers discussed above, delayers based on RF MEMS technology have the advantages of low power consumption, superior linearity, small size, low cost, and easy monolithic integration [5]. Therefore, in recent years, RF MEMS delayers have been extensively studied by both domestic and international scholars.

There are several classifications of MEMS delayers, such as distributed transmission line delay [6], reflective delay [7], and switched linear delay (TTDL) [8]. However, published literature and already-released TTDL products typically suffer from low device operating bands, low accuracy, and high loss. In 2007, Chu et al. of the University of Southern California proposed a path-sharing true-delay structure [9], with a design bandwidth of 1–15 GHz, a delay resolution of 15 ps, and a maximum delay of 225 ps. The chip adopts an 8-layer CMOS process, and its size is 3.1 mm × 3.2 mm. In 2013, Park et al. of Koryo University designed and fabricated a 15–40 GHz three-dimensional CMOS real-time delay (TTD) circuit with a maximum group time delay of 40 ps but with insertion loss as steep as 14 dB [10]. In 2019, a novel structure to achieve broadband true delay was proposed and implemented [11]. There is a maximum delay of 109.3 ps in the 8–18 GHz band and an average insertion loss of 18.2–22.5 dB in the expected band with an error of less than 4 ps.

This paper proposes an RF MEMS delay device with high delay accuracy and low loss in the Ka-band. The MEMS delay adopts the structure design of a single-blade multi-throw switch equipped with a linear time delay unit, which is characterized by a moderate chip area and an easily machinable structure. In addition, conventional switch delayers often use a cascade structure [12,13], with a minimum of 2n (n delay bits) switches driven for delay operation, and only two switches need to be driven to activate a delay state using the structure of this paper.

The paper is organized as follows. First, the development of a single-blade multi-throw switch (SPMT) is reviewed, and the designs of the upper electrode of the switch and the power divider are introduced in detail. Finally, the SP6T switch model designed in this paper is proposed, which has superior RF performance at DC-40 GHz. Then, a new delayed microwave line structure is presented, with innovations in transmission line corners and coplanar waveguide centerline for better delay performance. Finally, the proposed Ka-band five-bit MEMS delay, obtained by integrating two SP6T RF MEMS switches and a modern CPW microstrip, achieves elevated delay accuracy and low transmission loss.

## 2. Model Design

### 2.1. SP6T RF MEMS Switch Design

The SP6T RF MEMS switch is used as the core device to control the signal transmission path of the delay, and its RF performance has a great impact on the overall performance of the delay. The (SPMT) [14,15,16], developed so far, mainly has PIN diode class, RF coaxial class, and MEMS class. The listed PIN diode SPMT switches have the problems of narrower frequency band and larger size; the volume of RF coaxial SPMT switch developed by RF connector is also large; the MEMS SPMT switches still have the problems of poor isolation and insertion loss performance on the characteristics of small size and wide frequency band. To design the SP6T RF MEMS switch with good RF performance in Ka-band, this paper will optimize the upper electrode structure and power divider structure of the switch, respectively.

#### 2.1.1. Switch Upper Electrode Design

The proposed SP6T RF MEMS switch uses a series contact RF MEMS switch with a cantilever beam structure for the upper electrode of the switch, where one end of the upper electrode is fixed to the signal line by an anchor point and the remaining end is suspended above the contact. By applying a voltage to the driving electrode below the cantilever beam, the upper electrode is displaced by the electrostatic force and pulled down to contact with the contact, and the signal is conducted [17]. From the theory related to RF MEMS, it is known that the driving voltage for switch conduction [18] is:

(1) $$V=\sqrt{\frac{8k}{27\varepsilon_{0}A}}g_{0}^{3}$$

(2) $$k=\frac{Ewt^{3}}{4l^{3}}$$

where *k* refers to the elastic coefficient of the upper electrode of the switch; *E* means Young’s modulus of the material; *w* is the width of the upper electrode; *l* indicates the length of the upper electrode; *t* denotes the thickness of the upper electrode; *A* denotes the positive actuation region of the upper and lower electrodes of the switch; *g*_0_ denotes the initial spacing between the upper and lower electrodes; *ε*_0_ denotes the relative dielectric constant of air. The above equation shows that the driving voltage is proportional to the elastic coefficient and inversely proportional to the driving area.

**Figure 1:**
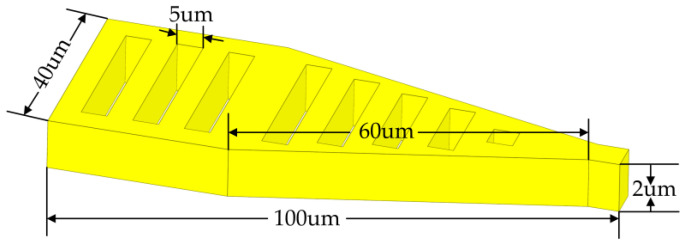
Triangular upper electrode structure and its specific parameters.

Most switches currently use a rectangular, straight-plate upper electrode [19]. Since it is more effective to reduce the cantilever beam elasticity coefficient than to increase the local driving area in reducing the driving voltage, a triangular upper electrode structure is used in this paper. The total length of the triangular upper electrode cantilever beam is 106 μm, consisting of a 40 μm rectangle, a 60 μm triangle, and a 6 μm rectangular block. Due to the skinning effect, 2 μm is chosen in this paper when designing the thickness of the upper electrode and CPW, which can ensure that the thickness of the metal is greater than 2 times the skinning depth at frequencies above 10 GHz, and reduce the metal resistance at low frequencies. The specific structure parameters are shown in Figure 1.

The proposed triangular upper electrode RF MEMS switch reduces the driving voltage in three main ways. First, by reducing the width of the cantilever beam to obtain a lower elasticity coefficient; second, by opening several openings in the upper electrode plate to reduce the air damping of the switch pull-down [20]; third, the hollowed-out triangular design significantly eliminates most of the switch mass when compared to the rectangular shape, thus reducing the weight of the switch. In addition, the opening of the hole in the upper pole plate facilitates the release of the sacrificial layer during process processing. The contact area between the triangular upper electrode structure and the transmission line is significantly smaller than the transmission line width compared to the straight upper electrode. A smaller contact area will reduce metal adhesion and provide better isolation performance.

**Figure 2:**
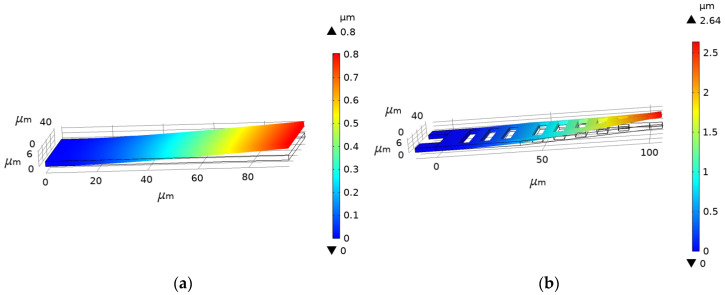
Schematic diagram of displacement of different structures under the same pressure (**a**) Schematic diagram of the displacement of a straight-plate structure; (**b**) Schematic diagram of the displacement of a triangular structure.

To verify that the optimized upper electrode structure is easy to pull down, the COMSOL software was used to apply equivalent pressure to both upper electrode structures, and the simulation results are shown in Figure 2. The displacement of the straight upper electrode is 0.8 μm, and that of the displacement of the triangular upper electrode is 2.64 μm. The results show that the triangular upper electrode structure is easier to pull down than the straight one. Using the above equations, the equivalent elastic coefficient of the cantilever beam was calculated to be 5.32 N/m, and the driving voltage of the triangular upper electrode was 13 v.

**Figure 3:**
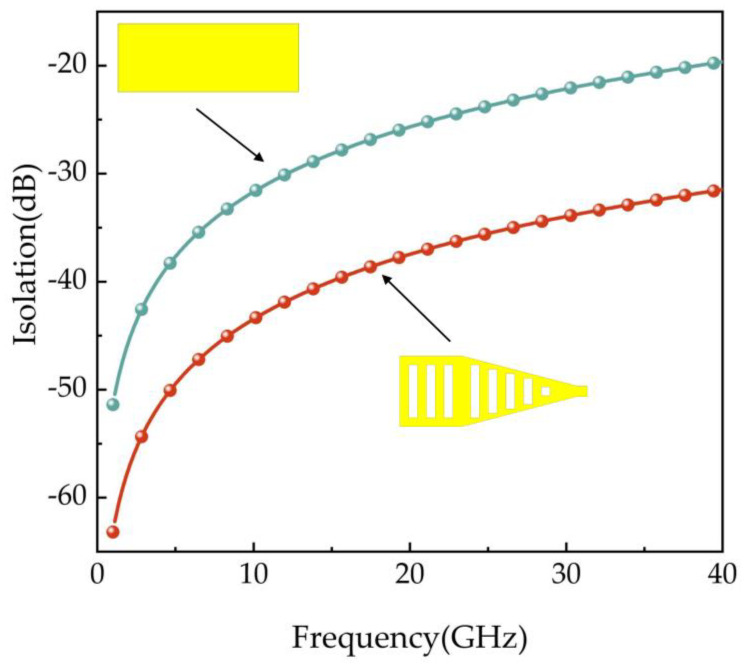
Isolation performance of different upper electrode structures.

To verify that the triangular upper electrode structure can improve the switching isolation, ANSOFT HFSS software was used to simulate and compare the switches of the two structures, the results of which are shown in Figure 3. It can be seen that the triangular upper electrode structure effectively improves the switching isolation in the Ka-band, with an improvement of 11.86 dB at 40 GHz and better isolation of 31.46 dB in the full frequency band.

#### 2.1.2. Switch Power Divider Design

**Figure 4:**
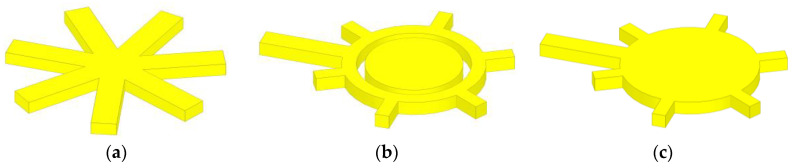
(**a**) General six-out-of-one power divider; (**b**) Circular power divider; (**c**) Disc-type power divider.

The function of the power divider (power splitter) is to proportionally distribute the input signal into the end branches [21], and its performance has an important impact on the performance of the SP6T RF MEMS switch in terms of insertion loss and signal splitting. The design expects to design a power divider structure to make the RF performance of each port of the SP6T RF MEMS switch consistent, so three power divider design models are proposed in this paper, as shown in Figure 4. Figure 4a is a general six-out-of-one power divider, whose structure is designed to divide into six branches of 100 μm length directly at the signal shunt. Figure 4b is a circular power divider, whose structure is designed to connect a 10 μm wide ring at the signal shunt and set a 75 μm block in the center of the circle as a ground wire. Figure 4c shows the disc-type power divider, whose structure is designed to set a 100 μm radius disc at the signal shunt.

**Figure 5:**
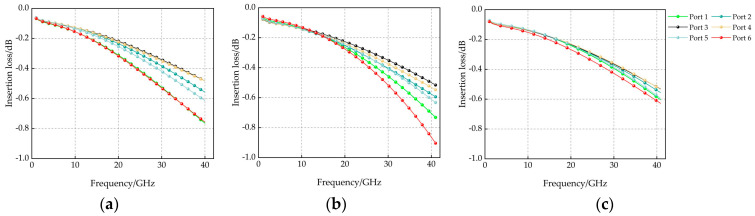
Insertion loss simulation results for different power divider structures: (**a**) General six-out-of-one power divider; (**b**) Circular power divider; (**c**) Disc-type power divider.

Next, the three power dividers and switches are cascaded, and their insertion loss performance was simulated, and the results are shown in Figure 5. A previous article verified that the closer the SPMT switch output is to the input, the stronger the electric field strength at its port, resulting in greater signal loss [22]. A look at the data in the three result plots shows that the closer the channel is to the input, the worse the insertion loss results for channel conduction, which is in line with the above theory. Because of the symmetrical geometry of the power divider, the insertion loss performance of Port 1 is similar to that of Port 6, Port 2 is similar to that of Port 4, and Port 3 is similar to that of Port 5.

From the perspective of signal loss, the insertion loss of the disc-type power splitter structure is the smallest at 40 GHz, and the maximum loss is only 0.62 dB. From the perspective of port performance consistency, the difference in insertion loss performance between the ports of the general six-out-of-one power divider at 40 GHz is 0.28 dB, the difference in insertion loss performance between the ports of circular-type power splitters is 0.39 dB, and the difference in insertion loss performance between the ports of disc-type power splitters is only 0.094 dB. On balance, the disc-type power splitter design best meets the RF performance requirements of this paper.

**Figure 6:**
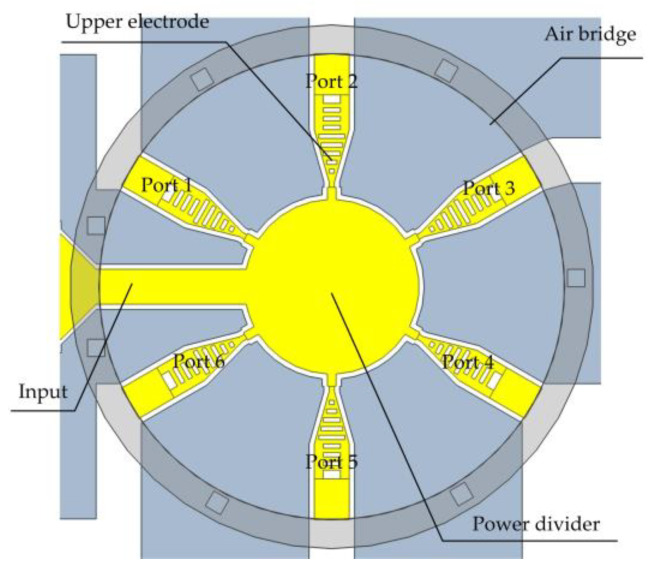
SP6T RF MEMS switch model.

The SP6T RF MEMS switch obtained by integrating the single-throw switch and the disc power divider is shown in Figure 6. The overall size of the switch is less than 0.6 mm × 0.6 mm, and the insertion loss of each port is less than 0.62 dB in Ka-band, with excellent overall RF performance.

### 2.2. Delay Structure Design

#### 2.2.1. CPW Transmission Line Corner Design

MEMS delayers are used to select different lengths of transmission lines through switches to achieve a multi-bit delay function. The formula for calculating the delay amount of the same microwave transmission line structure is as follows [23]:

(3) $$∆\varphi =\frac{2\pi f\sqrt{\varepsilon_{eff}}}{c}(l_{d}-l_{r})$$

**Figure 7:**
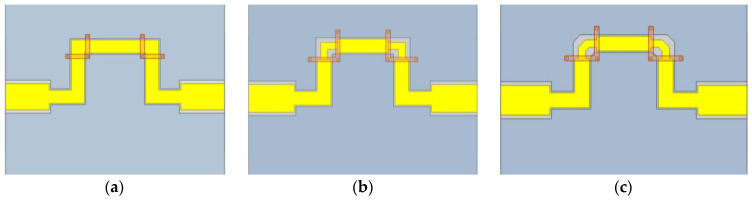
(**a**) Zigzag microstrip line layout; (**b**) Step compensation design; (**c**) Step compensation cut-angle design.

*c* is the free-space velocity; *ε_eff_* is the relative permittivity of the CPW equivalent; *f* is the operating frequency of the delay; *l_d_* is the delay transmission line length; *l_r_* is the reference line length. Equation (3) shows that the amount of delay is proportional to the length of the transmission line. To reasonably use the limited area of the substrate at the same time, as often as possible to achieve a larger delay, here is the use of zigzag microstrip line wiring, as shown in Figure 7a. Due to the CPW discontinuity at the corner, it is very easy to produce slot line mode, which is usually suppressed by erecting an air bridge to connect the ground lines on both sides [24].

To further reduce the transmission loss, the geometric structure at the corner of the microstrip line is optimally designed in this paper. As shown in Figure 7b,c, the step compensation design and step compensation tangent design are carried out at the corners, respectively.

**Figure 8:**
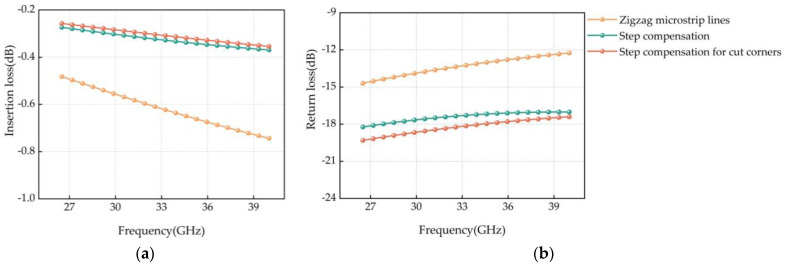
(**a**) Insertion loss simulation results for different corner designs; (**b**) Simulation results of echo resistance in different corner designs.

The simulation results of the transmission characteristics of different designs at the corner of the curved microstrip line are shown in Figure 8. From the simulation results, it seems that the RF performance of the design with step-compensated corner-cutting at the Ka-band is significantly improved, the insertion loss is improved by 0.39 dB at 40 GHz, and the return loss is improved by 5.16 dB at 40 GHz. Therefore, the design of step-compensated corner-cutting at the corner of the curved microstrip line is used.

**Figure 9:**
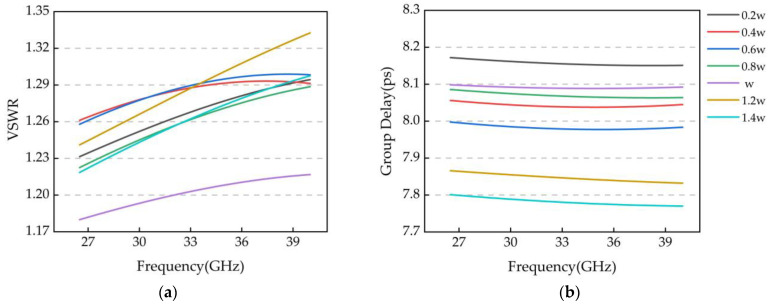
(**a**) Effect of angle of tangency on standing waves; (**b**) Effect of angle of tangency on the amount of delay.

For the corner-cutting design, there is an effect of the corner-cutting width d on the transmission characteristics of the zigzag microstrip line. The simulation results of the RF performance of the zigzag microstrip line with different cut-angle widths are shown in Figure 9, where w = 30 μm is the corner transmission line width. From Figure 9, it can be seen that different corner-cutting methods have a considerable impact on the standing wave and delay of the microstrip line. From the simulation results, with the increase of the length of the corner-cutting line (d = 0.2–1.4 W), the delay performance of the transmission line deteriorates progressively, and the amount of the delay decreases by nearly 0.39 ps; at the same time, the VSWR obtained when the length of the corner-cutting line is d = w is the best, and it is optimized by 0.12 relative to that of d = 0.2 w. Therefore, as a compromise, the width of the tangent angle with d = w is chosen.

#### 2.2.2. Coplanar Waveguide Loading Gap Design

**Figure 10:**
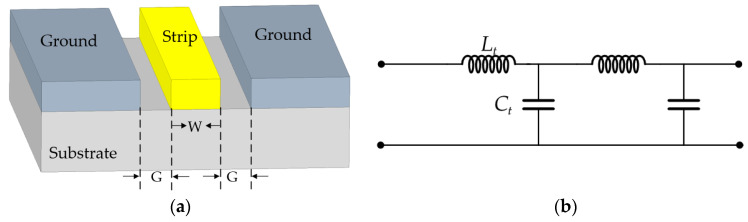
(**a**) Structure of Coplanar Waveguide; (**b**) CPW equivalent circuit.

In this paper, a coplanar waveguide structure is used for signal transmission, as shown in Figure 10a, and its equivalent circuit is shown in Figure 10b [25], where the equations for the unit capacitance *C_t_* and unit inductance *L_t_* in the circuit are Equations (4) and (5), respectively, where *c* is the velocity in free space, *Z*_0_ is the characteristic impedance of the unloaded transmission line, and *ε_eff_* is the effective dielectric constant of the CPW.

The coplanar waveguide structure achieves the delay by controlling the phase velocity *v* of the transmission line, as shown in Equation (6), where the magnitude of the phase velocity depends on the unit capacitance *C_t_* and the unit inductance *L_t_*.

(4) $$C_{t}=\frac{\sqrt{\varepsilon_{eff}}}{cZ_{0}}$$

(5) $$L_{t}=C_{t}Z_{0}^{2}$$

(6) $$v=\sqrt{\frac{1}{L_{t}(\frac{C_{t}}{G})}}$$

**Figure 11:**
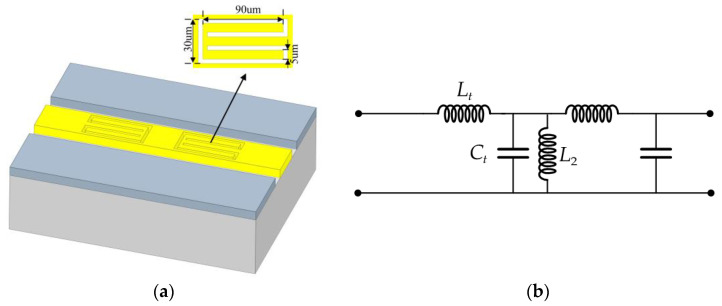
(**a**) Coplanar waveguide loading gap structure; (**b**) CPW loads a gap equivalent circuit.

To increase the delay amount without increasing the length of the delay line, this paper improves the unit inductance of the delay line by replacing the common transmission line with a coplanar waveguide loading gap, thus increasing the phase speed and increasing the delay amount. A CPW discontinuous transmission structure was previously proposed by Tang et al. [26], in which two rectangular slots are hollowed out in the central signal line of the CPW, which forms another coplanar waveguide in the central guide member. According to transmission line theory, this structure is equivalent to a short circuit along the propagation direction and can be equated to a parallel inductor. The results of the article show that this CPW discontinuous structure design can not only widen the operating band but also keep the insertion loss low while obtaining a large phase shift. Therefore, this paper proposes a structure that adds a U-shaped slit to the center guide of the coplanar waveguide to equivalently form three coplanar waveguides on the center guide, thus achieving the purpose of introducing a larger equivalent inductance *L_2_*. The specific structure of the coplanar waveguide loading gap is shown in Figure 11a, and the equivalent circuit is shown in Figure 11b.

**Figure 12:**
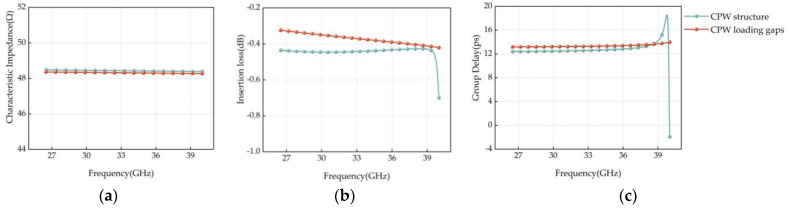
(**a**) characteristic Impedance simulation comparison; (**b**) Insertion loss simulation comparison; (**c**) Simulation comparison of time delay.

The RF performance simulations of the normal CPW structure and the loaded U-shaped slit CPW structure were performed using ANSOFT HFSS software, and the comparison results are shown in Figure 12. As shown in Figure 12a, the characteristic impedance of the CPW center guide is simulated before and after loading the U-shaped slit, and the result is that the introduction of the surface U-shaped slit has a small impact on the characteristic impedance of the transmission line, which only changes 0.11 Ω at 40 GHz. As the characteristic impedance of RF devices is generally 50 Ω, the final determination of the model signal input and output port width is 120 μm, the center guide and both sides of the ground spacing is 16 μm. As can be seen in Figure 12b,c, the RF performance of the common CPW structure deteriorates sharply at the high operating frequency band of 39.78 GHz. Comparatively, the CPW-loaded U-shaped slit outperforms the normal CPW structure in terms of insertion loss performance and delay performance in the full frequency band 26.5–40 GHz. In terms of insertion loss performance, the CPW-loaded U-shaped slit structure reduces 0.22 dB on average over the full frequency band. In terms of delay performance, the CPW-loaded U-shaped slit structure not only increases the delay by an average of 0.78 ps in the whole frequency band but also provides better delay flatness. In summary, the CPW load gap not only does not have a greater impact on the characteristic impedance of the transmission structure but also reduces the insertion loss, improves the delay amount and delay flatness, and is more suitable for the delay transmission structure in this paper.

### 2.3. Five-Bit Delay Model Design

**Figure 13:**
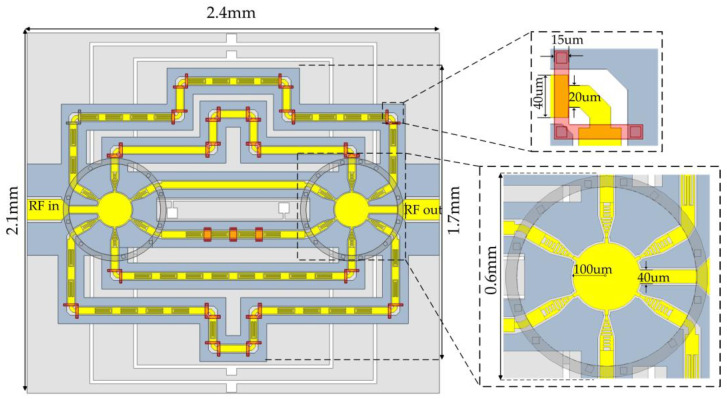
Model diagram of a Ka-band five-bit MEMS delay.

**Figure 14:**
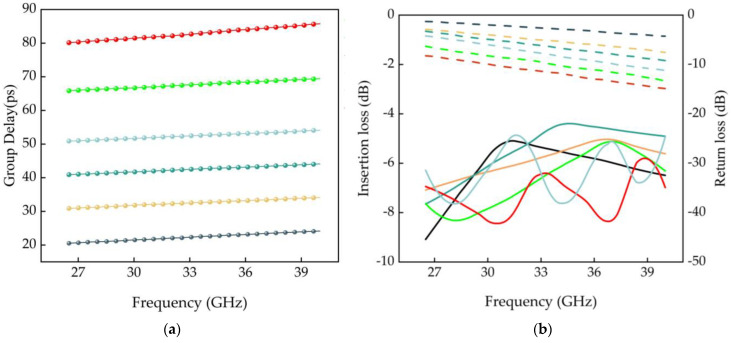

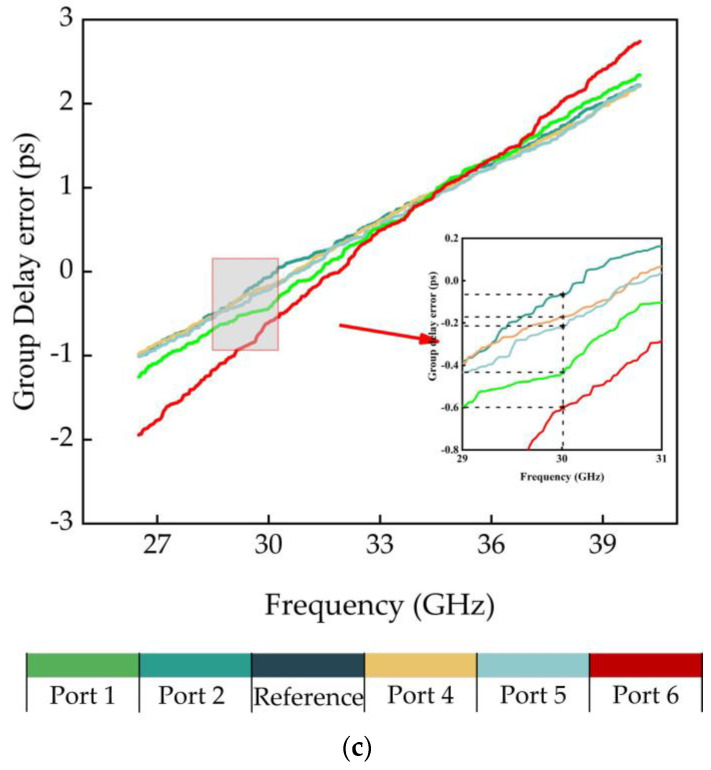
(**a**) Device delay simulation results; (**b**) Simulation results of device insertion loss and return resistance; (**c**) Delay error.

Determined by the above structure design, a five-bit MEMS delayer for an integrated modeling simulation model is shown in Figure 13, with an overall size of 2.1 mm × 2.4 mm × 0.5 mm. The model delay center frequency is 30 GHz, and the simulation results of the RF performance are shown in Figure 14 and Table 1. The simulation results of the five-bit MEMS delayers show that the error of the delay amount at the center frequency of 30 GHz is less than 0.63 ps, and the return loss is better than 28 dB. In the whole operating band (26.5–40 GHz), the delay error of all states is within 22.2%, the return rejection is better than 21.54 dB, and the insertion loss is less than 3.69 dB. The results show that the overall performance of the device is superior, although the delay accuracy and delay error increase with the increase of delay amount when each delay unit works individually in the five-bit MEMS delay.

## 3. Comparison and Discussion

Table 2 shows the performance comparison of the five-bit MEMS delay designed in this paper and the multi-bit delay studied by various institutions in recent years. As shown in Table 2, the main process used in the current multi-digit delay design is the CMOS technology. Comparison of this paper with other published results shows that the multi-bit delay timers fabricated by the pseudomorphic HEMT (PHEMT) process and CMOS process have the advantages of small device size but suffer from the problems of low bandwidth and high insertion loss. Compared to the five-bit MEMS delay designed in this paper, which is close to the multi-delay made by the CMOS technology in terms of device size, and in terms of RF performance has the advantages of a high-frequency band, low loss, and high accuracy, and the device has a high potential for application in Ka-band.

## 4. Conclusions

A five-bit MEMS delay device integrated by a symmetric SP6T RF MEMS switch and a CPW microstrip line is proposed. The device is innovatively designed with a triangular upper electrode structure, disc-type power divider, a step-compensated tangent structure at the corner, and a coplanar waveguide loaded U-shaped slit structure to achieve 0–60 ps delay on an area of 2.1 mm × 2.4 mm. Simulation results show this design has high delay accuracy at the 30 GHz frequency point and superior return loss and insertion loss performance in the frequency band of 26.5–40 GHz. Compared with other reported delayers, the optimization method proposed in this paper can provide a new idea for the design of delayers operating in high-frequency bands. However, this paper’s MEMS five-bit delay research is only for theoretical modeling, which can be used in practice in the radio frequency system, but also needs to be modeled through the MEMS process technology to process the finished product after measurement debugging. Therefore, future work in this paper will focus on device process research.

## Figures and Tables

**Table 1:** Simulation performance of a five-bit delay.

State	Target	Delay (ps)				Return Loss
(bit)	(ps)	Center	Subtraction	Accuracy	Error	(dB)
0	0	21.50				−28.68
1st	10	31.44	9.94	±2.22	−0.06	−31.74
1st	10	31.44	9.94	±2.22	−0.06	−31.74
2nd	20	41.33	19.83	±2.21	−0.17	−30.60
3rd	30	51.29	29.79	±2.20	−0.21	−39.19
4th	45	61.07	44.57	±2.34	−0.43	−40.71
5th	60	80.9	59.40	±2.54	−0.6	−31.20

**Table 2:** Comparison of delay in recent years.

Ref	Frequency	Bit	Delay	Max Error	Insertion Loss	Technology	Size
	(GHz)		(ps)	(ps)	dB		mm
2001 [27]	0–40	3	86	3	4.3	MEMS	5 × 6
2007 [9]	1–15	4	255	15	-	CMOS	3.1 × 3.2
2013 [10]	15–40	3	40	5	14	CMOS	1.1 × 0.9
2018 [28]	6–18	8	255	29	-	CMOS	2.0 × 0.6
2019 [11]	8–18	3	109.3	4	22.5	CMOS	0.9 × 2.1
2021 [29]	8–18	5	120	3.9	20.5	CMOS	1.2 × 2.7
2022 [30]	0–0.8	4	3800	4	-	CMOS	1.45 × 1.37
2023 [31]	6–18	3	106	10	15	PHEMT	2.7 × 0.73
This work	26.5–40	5	60	2.54	3.69	MEMS	2.1 × 2.4

## Data Availability

Not applicable.
